# Antimicrobial Resistance and Biofilm Formation in Coagulase-Negative *Staphylococcus* and *Mammaliicoccus* spp. from Poultry Meat in Spain

**DOI:** 10.3390/microorganisms14061195

**Published:** 2026-05-26

**Authors:** Paula Eguizábal, Rocío Lopéz-Saenz de Navarrete, Rosa Fernández-Fernández, Carmen González-Azcona, Allelen Campaña-Burguet, Irene Marañón-Clemente, Tamara Álvarez-Gómez, Paula Corral-Zorzano, Daniel Benito, Carmen Torres, Carmen Lozano

**Affiliations:** Area of Biochemistry and Molecular Biology, OneHealth-UR Research Group, University of La Rioja, 26006 Logroño, Spain; paula.eguizabalm@unirioja.es (P.E.); rosa.fernandez@unirioja.es (R.F.-F.); carmen.gonzalezaz@unirioja.es (C.G.-A.); allelencampanaburguet@gmail.com (A.C.-B.); irene-olga.maranon@unirioja.es (I.M.-C.); tamara.alvarez@unirioja.es (T.Á.-G.); paula.corral@unirioja.es (P.C.-Z.); daniel.benito@unirioja.es (D.B.)

**Keywords:** coagulase-negative *Staphylococcus*, *Mammaliicoccus*, antimicrobial resistance, biofilm formation, poultry meat

## Abstract

Coagulase-negative staphylococci (CoNS) and mammaliicocci (MA) are common in food-derived samples and may act as antimicrobial resistance (AMR) reservoirs. A previous study reported a high *S. aureus* prevalence in poultry meat. The objective of this study was to characterize the species diversity, antimicrobial resistance, and biofilm-forming capacity of CoNS/MA from the same food samples. Species identification, antimicrobial susceptibility testing, resistance gene detection, molecular typing, and biofilm formation assays were performed. One hundred and forty-eight non-repetitive CoNS/MA isolates were detected in 85% of samples, and 14 species were identified. The most prevalent species were *S. epidermidis* (18.2%), *S. simulans* (12.8%), *S. saprophyticus* (12.2%), *S. warneri* (11.5%), and *M. lentus* (10.1%). Most samples harbored one or two different species, although some showed higher diversity. Although 27.0% of isolates were pan-susceptible, 22.3% were multidrug-resistant (MDR), significantly associated with *M. lentus* and *S. epidermidis*. Methicillin resistance was found in 10 isolates, mainly in *S. epidermidis* (lineages ST9, ST59, ST88 and ST640). Biofilm formation was observed in 24.3% of isolates (some of them MDR) and was significantly associated with *S. pasteuri* and *S. xylosus* and with samples from supermarkets. No methicillin-resistant isolates were biofilm producers. These findings highlight the diversity of CoNS/MA in poultry meat and their role as AMR reservoirs and persistence factors, emphasizing their relevance in food safety.

## 1. Introduction

Foodborne diseases remain one of the biggest global challenges nowadays. About 600 million people suffer foodborne illnesses each year, according to the World Health Organization [1]. Forty percent of them affect children under 5 years of age, with 125,000 deaths each year [2]. Moreover, the food chain is increasingly recognized as a key route for the dissemination of antimicrobial-resistant bacteria and antimicrobial resistance genes, reinforcing the need for continuous surveillance of food products [3].

In this sense, poultry meat represents a particularly relevant matrix. It is one of the most widely consumed animal-derived foods worldwide and can harbor complex bacterial communities originating from the animal microbiota, the slaughter environment, and subsequent processing and retail stages [3,4,5]. Within these communities, *Staphylococcus* spp. can be frequently detected along the poultry production chain and in retail meat, making poultry meat an important interface for the transmission and persistence of staphylococci and associated resistance determinants [6].

The genus *Staphylococcus* is traditionally divided into coagulase-positive *Staphylococcus* (CoPS) and coagulase-negative *Staphylococcus* (CoNS) based on their ability to coagulate blood plasma. The main representative of CoPS is *S. aureus*, which is one of the major causes of foodborne intoxications worldwide due to the secretion of heat-stable staphylococcal enterotoxins [7], as well as an important hospital and community pathogen [8]. Consequently, *S. aureus* has historically been the primary focus of staphylococcal research in both clinical and food safety contexts.

In contrast, CoNS were long regarded as commensal bacteria lacking the classical virulence factors of *S. aureus*. However, this view has changed substantially in recent years due to the emergence of nosocomial infections caused by CoNS. *S. epidermidis* is the most studied CoNS species because it is frequently associated with clinical infections [9,10]. Other well-investigated species are *S. lugdunensis*, *S. saprophyticus*, *S. haemolyticus*, *S. caprae* and *S. capitis* due to their content of virulence factors [11]. In addition to the clinically relevant species, other CoNS—including *S. xylosus*, *S. equorum*, and *S. warneri*—have been frequently isolated from food and food-processing environments [12], highlighting their relevance beyond the clinical setting.

Very recently, according to Madhaiyan et al. (2020), five former *Staphylococcus* species were reclassified into the new genus *Mammaliicoccus* (MA), which is phylogenetically close to *Staphylococcus* [13]. Members of this group, including *M. lentus* and *M. sciuri*, are widely distributed in animals, humans and environmental reservoirs and are increasingly detected in food-associated samples [14].

In terms of antimicrobial resistance (AMR), CoNS and MA deserve particular attention. Although many isolates remain susceptible to commonly used antimicrobials, others have accumulated a wide range of resistance determinants following exposure to antimicrobial selective pressures in animal production, veterinary settings and the environment. In this regard, the detection of methicillin-resistant coagulase-negative *Staphylococcus* (MRCoNS) or methicillin-resistant *Mammaliicoccus* (MRMA) is a growing concern due to their potential role as reservoirs and vehicles for the dissemination of clinically relevant antimicrobial resistance determinants, including *mecA*, through horizontal gene transfer [15]. This role is especially relevant in food-associated contexts, where resistant commensal bacteria may contribute to the wider spread of antimicrobial resistance within the food chain.

Moreover, another important characteristic of CoNS isolates is their potential ability to produce biofilms. Biofilms are bacterial communities embedded in a self-produced extracellular polymeric matrix [16]. They provide bacteria a protective environment, increasing antibiotic resistance and facilitating chronic infections, as well as protecting the bacteria from host immune responses [17]. In addition to the clinical relevance of the biofilm, the persistence of foodborne pathogens within these structures on food-contact surfaces can compromise the quality and safety of food products [18].

A previous study carried out in our research group reported a high prevalence of *S. aureus* in food samples of poultry origin [19]. During that investigation, a large number of CoNS and MA isolates were also recovered from the same samples, but were not characterized [19]. Given the increasing recognition of CoNS and MA as potential reservoirs of antimicrobial resistance and persistence-associated traits, together with the limited information available regarding their occurrence in retail poultry meat, the objective of the present study was to identify the CoNS and MA species present in these chicken meat samples and to characterize their antimicrobial resistance phenotype and genotype, as well as their biofilm-forming ability.

## 2. Material and Method

### 2.1. Bacterial Identification

A total of 186 CoNS and MA isolates (146 and 40, respectively) were recovered from 60 samples of chicken meat analyzed in a previous study [19] and were included in this study for further characterization. All 60 samples were of chicken meat intended for human consumption, including chicken breasts and thighs. Twenty-eight samples were obtained from supermarkets and 32 from butcher retail shops, all located in La Rioja (Spain).

As previously described [19], up to six colonies per plate showing staphylococcal morphology were selected and identified by MALDI-TOF mass spectrometry (matrix-assisted laser desorption-ionization time-of-flight) using the standard protein extraction protocol recommended by the manufacturer (Bruker Daltonik, Bremen, Germany).

### 2.2. Antimicrobial Resistance Phenotype and Genotype

The susceptibility to 11 antibiotics was tested by the disk diffusion method in all 186 isolates. The antibiotics studied were the following ones (µg/disk) (Oxoid, Basingstoke, UK): penicillin (10), cefoxitin (30), erythromycin (15), clindamycin (2), gentamicin (10), tobramycin (10), tetracycline (30), ciprofloxacin (5), chloramphenicol (30), linezolid (30), and trimethoprim-sulfamethoxazole (1.25 + 23.75). The disk diffusion results were interpreted according to the European Committee of Antimicrobial Susceptibility Testing [20]. For ciprofloxacin, isolates with inhibition zone values above the susceptible breakpoint but below the resistance breakpoint were considered susceptible category. The isolates that presented resistance to at least 3 different antibiotic families were considered multidrug-resistant isolates (MDR). *M. sciuri* has an intrinsic mechanism of clindamycin resistance (mediated by *sal*(A) gene); therefore, this antibiotic was excluded from the MDR classification and from resistance phenotypes in this species. The isolates that showed susceptibility to all tested antibiotics were considered pan-susceptible.

After the identification was performed and the antibiotic susceptibility was studied for the 186 isolates, three criteria were established to determine which isolates were non-repetitive: (a) isolates obtained from different samples; (b) isolates from the same sample but belonging to different species; and (c) isolates from the same sample and species that exhibited different antibiotic resistance phenotypes.

Resistance mechanisms were examined according to the antibiotic resistance patterns observed in non-repetitive isolates. The following genes were studied by PCR for each antibiotic or antibiotic family: beta-lactams (*mecA*, *mecC*, and *blaZ*), erythromycin and/or clindamycin [*erm*(A), *erm*(B), *erm*(C), *erm*(T), *msr*(A), *mph*(C), *lnu*(A), *lnu*(B), *lsa*(B), and *vga*(A)], gentamicin-tobramycin [*aac*(6′)-Ie-*aph*(2″)-Ia, *ant*(4′)-Ia], and trimethoprim-sulfamethoxazole (*dfrA*, *dfrD*, *dfrG* and *dfrK*) [11]. Positive and negative controls from the University of La Rioja were included in each PCR run.

### 2.3. Molecular Typing of Methicillin-Resistant S. epidermidis and MRCoNS/MA

Multilocus sequence typing (MLST) was performed for all methicillin-resistant *S. epidermidis* isolates (MRSE). The seven housekeeping genes (*arcC*, *aroE*, *gtr*, *pyrR*, *mutS*, *tpi*, and *yqiL*) were amplified by PCR and sequenced by Sanger sequencing to determine the Sequence Type (ST) [21].

### 2.4. Biofilm Assay

Bacterial biofilm formation was investigated as described by Schwartbert et al., with some modifications [22]. Overnight cultures of *Staphylococcus* spp. and *Mammaliicoccus* spp. were diluted 1:200 in BHI (Condalab, Madrid, Spain) supplemented with 0.25% glucose (PanReac AppliChem, Barcelona, Spain) and 4% NaCl (PanReac AppliChem, Barcelona, Spain). From this dilution, 200 µL was added to the wells of a 96-well polystyrene microtiter plate (Greiner Bio-One, Frickenhausen, Germany) and incubated for 24 h at 37 °C. All plates were washed twice with 200 µL PBS per well, and the biofilms were stained with 100 µL of 0.1% crystal violet (PanReac AppliChem, Barcelona, Spain) per well for 15 min at room temperature. After 2 additional washing steps with PBS (Gibco, Grand Island, NY, USA), biofilms were solubilized in 100 µL ethanol–acetone (80:20). The absorbance was measured at 655 nm using a microtiter plate reader (Spark 10M, Tecan, Männedorf, Switzerland). *S. epidermidis* RP62A was used as the positive control and *S. carnosus* TM300 as the negative control. Each isolate was studied in three biological replicates and in eight wells per microtiter plate. The percentage of biofilm biomass was calculated relative to the biofilm production of the reference *S. epidermidis* RP62A isolate. Five categories were established according to the percentage of biofilm formation: no biofilm, <25%; weak, 25–50%; moderate, 50–75%; strong, 75–100%; and very strong, >100%.

### 2.5. Statistical Analysis

Data from non-repetitive CoNS and MA isolates were analyzed. The association between the origin (supermarket or butcher retail shops) and the species, methicillin resistance, MDR phenotype, susceptibility phenotype, and biofilm formation capacity was evaluated. The possible association between antibiotic resistance and biofilm formation was also evaluated. In addition, the possible association between the species and the detection of MDR isolates, methicillin-resistant isolates or biofilm-producing isolates was analyzed. For that, Pearson’s Chi-square test or Fisher’s exact test (when sample sizes were too small) was used. To identify which species differed significantly from the others, post hoc comparisons were performed using Fisher’s exact test. Moreover, a potential association between species was investigated. Pairwise associations between species were assessed using Fisher’s exact test to account for small sample sizes and low counts and to control for multiple comparisons across all species pairs. *p*-values were adjusted using the Benjamini–Hochberg method and odds ratios (OR), which were calculated to estimate the direction and magnitude of association. The presence of *S. aureus* described in previous reports in the same samples [19] was used in this analysis. All statistical analyses were performed with R (v 3.6.0+) and RStudio (v 2025.09.1+401) software. Results were considered statistically significant at *p* < 0.05.

## 3. Results

### 3.1. Selection of Non-Repetitive Isolates

A total of 186 isolates were obtained from 60 poultry meat samples. CoNS and MA were detected in 51 samples (85%). After identification and antimicrobial phenotype determination, 148 non-repetitive isolates were further studied. For all the results presented below, these 148 isolates were considered.

### 3.2. Species Identification

A total of 14 different species of CoNS and MA were detected, with 10 of them being CoNS and 4 MA (Table 1). The most common species were *S. epidermidis* (18.2%), *S. simulans* (12.8%), *S. saprophyticus* (12.2%), *S. warneri* (11.5%), and *M. lentus* (10.1%). The remaining species showed a frequency of less than 10%. No significant association was observed between species distribution and sample origin after correction for multiple comparisons (*p*_adj > 0.05 for all species).

### 3.3. Intra-Sample Species Diversity

**Figure 1:**
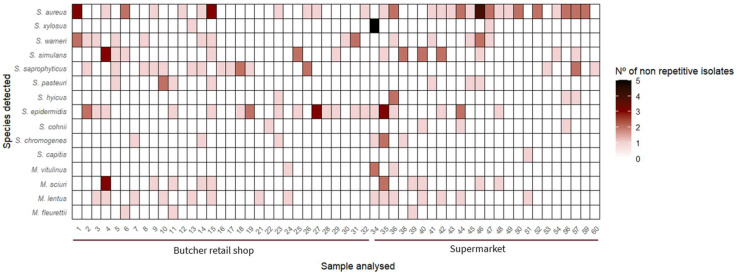
Distribution of the 148 non-repetitive isolates according to their species and type of sample.

At least one CoNS or MA isolate was detected in 51 out of 60 poultry meat samples analyzed (Figure 1). Most of the samples harbored only one or two CoNS or MA species (Figure 1). However, in four samples (samples 15, 34, 35, 36), a high species diversity was observed, with five to six distinct CoNS/MA species identified, and three to four species were detected in 12 samples. In six samples only *S. aureus* was detected, whereas in 22 it was identified in combination with other CoNS and/or MA.

Statistical analysis of co-occurrence between *S. aureus* and the other 14 species revealed no significant associations after correcting for Benjamini–Hochberg (*p*_adj > 0.05). Although some species such as *S. saprophyticus*, *S. xylosus*, *M. vitulinus*, and *M. fleurettii* showed OR < 1 compared to *S. aureus*, suggesting a tendency not to co-occur, these differences were not statistically significant after correction for multiple comparisons.

Other species, including *S. simulans*, *S. warneri*, *M. sciuri*, *S. pasteuri*, and *S. epidermidis*, had OR > 1, suggesting a slight tendency to co-occur with *S. aureus*, although these trends were also non-significant. Pairwise comparisons among the remaining species revealed that only *S. warneri* and *S. pasteuri* co-occurred significantly within the same samples (*p* = 0.0082), while most other species pairs showed weak or non-significant associations.

### 3.4. Antimicrobial Resistance Phenotype and Genotype

**Figure 2:**
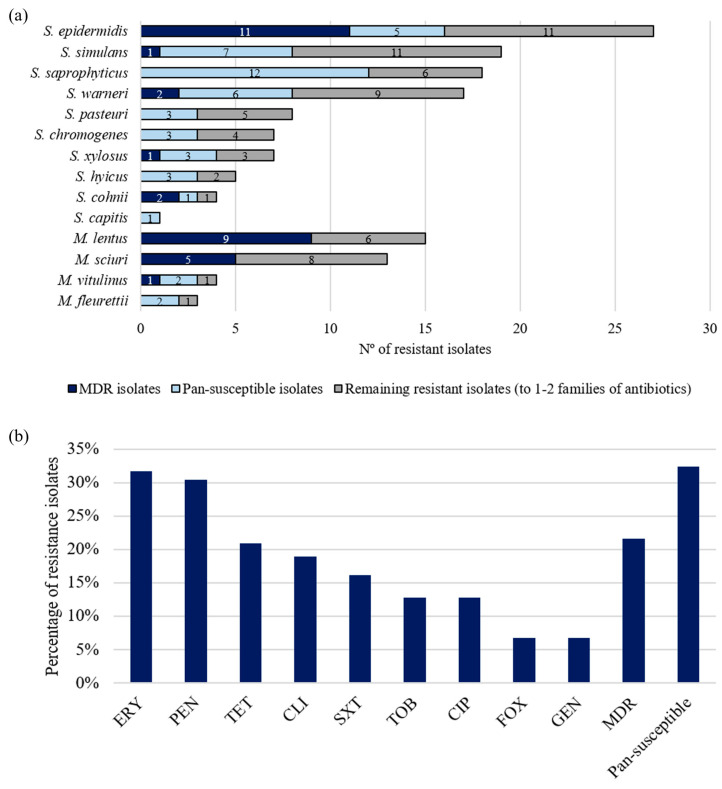
(**a**) Antimicrobial resistance phenotype in the different CoNS and MA species detected in 60 poultry meat samples. (**b**) Antimicrobial resistance rates detected in CoNS and MA isolates. PEN, penicillin; FOX, cefoxitin; ERY, erythromycin; CLI, clindamycin; TET, tetracycline; GEN, gentamycin; TOB, tobramycin; SXT, trimethoprim-sulfamethoxazole; CIP, ciprofloxacin; MDR, multidrug-resistant; pan-susceptible: susceptible to all tested antibiotics.

Forty-eight isolates (32.4%) were susceptible to all the antibiotics tested (pan-susceptible), and forty-one isolates were resistant to only one antibiotic. Species distribution among pan-susceptible isolates (no. pan-susceptible/no. total for that species) was as follows: *S. epidermidis* (5/27), *S. simulans* (7/19), *S. saprophyticus* (12/18), *S. warneri* (6/17), *S. pasteuri* (3/8), *S. chromogenes* (3/7), *S. xylosus* (3/7), *S. hyicus* (3/5), *S. cohnii* (1/4), *M. vitulinus* (2/4), *M. fleurettii* (2/3), and *S. capitis* (1/1). Thirty-one isolates (22.3%) were MDR and belonged to 8 different species (*S. cohnii*, *S. epidermidis*, *S. simulans*, *S. xylosus*, *S. warneri*, *M. lentus*, *M. sciuri*, and *M. vitulinus*) (Figure 2a). Ten isolates were methicillin resistant: *S. epidermidis* (*n* = 6), *M. fleurettii* (*n* = 1), *S. hyicus* (*n* = 1), *M. lentus* (*n* = 1) and *M. sciuri* (*n* = 1) (Table 2).

The highest resistance percentages were for erythromycin (31.8%) and penicillin (30.4%) (Figure 2b and Table 2). All the isolates showed susceptibility to linezolid and chloramphenicol.

The antimicrobial resistance mechanisms detected for each antibiotic were (gene/nº of isolates): penicillin (*blaZ*/45), cefoxitin (*mecA*/10), erythromycin [*msr*(A)/35, *mph*(C)/24], erythromycin-clindamycin [*erm*(A)/15, *erm*(B)/4, *erm*(C)/10, *erm*(T)/1], clindamycin [*lnu*(A)/10, *vga*(A)/3, *sal*(A)/13], gentamicin-tobramycin [*aac*(6′)-Ie-*aph*(2″)-Ia/10], tobramycin (*ant*(4′)-Ia/16), tetracycline [*tet*(K)/28, *tet*(L)/26, *tet*(M)/9] and trimethoprim-sulfamethoxazole [*dfr*(A)/14, *dfr*(G)/6, *dfr*(K)/16] (Table 2).

After evaluating the association between the origin of the isolates and the antimicrobial resistance phenotype detected, no statistically significant association was observed (*p* = 0.7539 for methicillin resistance, *p* = 0.8751 for pan-susceptibility and *p* = 0.4717 for MDR). At the species level, a statistically significant association was detected between species and the MDR phenotype (*p* = 0.0005). *M. lentus* and *S. epidermidis* showed significantly higher proportions of MDR isolates compared to the other species. No significant global association was found between the methicillin resistance phenotype and species (*p* = 0.1029). Nevertheless, *S. epidermidis* showed a higher frequency of methicillin-resistant isolates than the other species.

### 3.5. Molecular Typing of Methicillin Resistance S. epidermidis

Four different STs were detected among the six methicillin-resistant *S. epidermidis *isolates. ST88 was detected in 2 isolates, ST640 in another 2 isolates, and the remaining two isolates were ST9 and ST59. The two ST640 isolates were obtained from supermarket samples, whereas the remaining MRSE isolates were recovered from butcher retail shops (Supplementary Figure S1).

### 3.6. Biofilm Formation Assay

**Figure 3:**
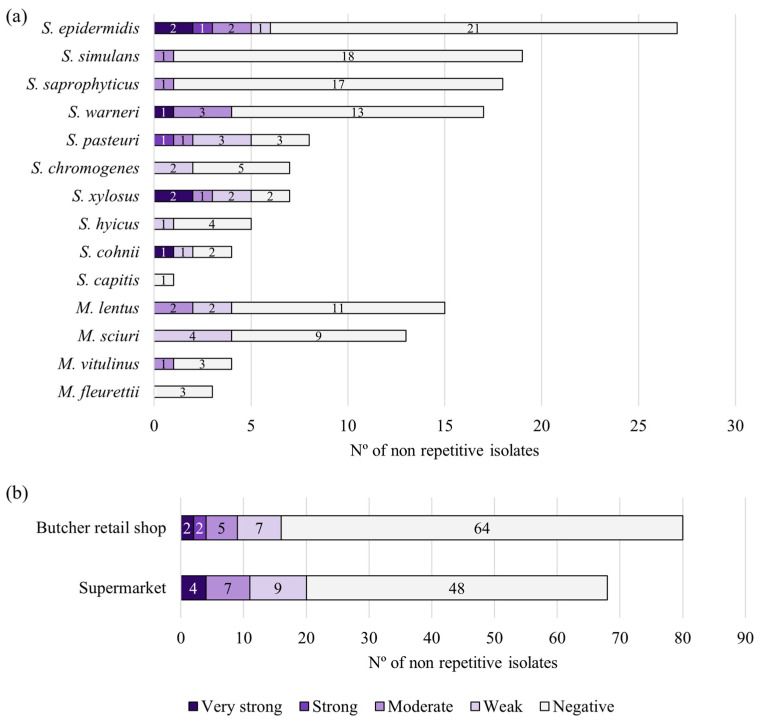
(**a**) Biofilm production in the different CoNS and MA species detected in poultry meat samples. (**b**) Biofilm production according to the origin of the samples in which the isolates were detected.

In this study, 24.3% of the isolates were biofilm producers. Among these, two isolates were strong producers and belonged to *S. epidermidis* (*n* = 1) and *S. pasteuri* (*n* = 1) species, and six isolates were considered very strong biofilm formers [*S. epidermidis* (*n* = 2), *S. xylosus* (*n* = 2), *S. cohnii* (*n* = 1), *S. warneri* (*n* = 1)] (Figure 3a and Table 2). Biofilm formation capacity was significantly associated with species (*p* = 0.0165), with *S. pasteuri* and *S. xylosus* showing higher proportions of biofilm producers than the other species (Figure 3a). None of the MRCoNS/MA were biofilm producers. Nine of the biofilm producers were also MDR isolates, these being *M. sciuri* (*n* = 3), *S. epidermidis* (*n* = 3), *S. cohnii* (*n* = 1), *S. warneri* (*n* = 1) and *S. xylosus* (*n* = 1). Moreover, two of these MDR and biofilm formers produced very strong biofilms (*S. epidermidis* and *S. xylosus*) (Table 2).

## 4. Discussion

This study analyzed 148 non-repetitive CoNS and MA isolates recovered from 60 retail chicken meat samples in Spain. The detection of CoNS or MA in 85% of samples is consistent with previous reports describing these organisms as common components of poultry-associated microbiota [23]. However, this prevalence is higher than that reported in other investigations, where detection rates ranged from 32% to 64% [6,24].

A wide diversity of species was observed, with *S. epidermidis*, *S. simulans*, *S. saprophyticus*, *S. warneri* and *M. lentus* being the most frequently detected (64.8% of the isolates). Similar species distributions have been reported in other studies in poultry samples, where *S. simulans* and *M. lentus* were commonly identified [6,25] or *S. epidermidis* in animal-derived food [17,26].

No significant associations were found in most co-occurrence analyses between species (except for *S. warneri* and *S. pasteuri*), suggesting a complex and structured microbial community rather than dominance by a single species. This pattern remained consistent even when considering the previously reported presence of *S. aureus* in the same samples [19], indicating that community composition is not driven by competitive exclusion by this classical opportunistic pathogen.

Although the presence of CoNS or MA in the food chain does not usually represent a direct risk to consumers, these species may act as reservoirs of antimicrobial resistance determinants that can be horizontally transferred to more virulent staphylococcal species [27]. In this context, the prevalence of resistance observed in this study (31.8% to erythromycin, 30.4% to penicillin) is comparable to reports from other European poultry studies [28,29]. The detection of MRCoNS/MA (6.8%) and MDR (22.3%) isolates underscores their epidemiological importance. The proportion of methicillin-resistant isolates in our study was lower than that reported in poultry-derived samples from Portugal [6] and Egypt [30] where the rates were 39–56%, but was similar to the 8% described in the United States [31] or the 16% reported in Poland [24].

Recent reviews emphasize that poultry products could act as critical nodes for AMR dissemination across domains [32]. Moreover, a recent study combining human and poultry isolates reported overlapping *mecA* lineages of *M. sciuri* and *S. epidermidis*, evidencing direct cross-sector transmission potential [33]. The detection of MRSE is particularly relevant considering that this species represents one of the leading causes of healthcare-associated infections, especially in bloodstream infections related to central venous catheters and surgical site infections, mainly affecting immunocompromised and critically ill patients [34]. The MRSE population identified in this study included multiple STs. Some of the detected STs (ST59, ST88 and ST640) have previously been described in food-related [35] or livestock-associated environments [36,37], as well as in hospital settings [34,38,39], suggesting a potential overlap between food, animal, and clinical reservoirs within a One Health context. In particular, ST88 has previously been identified in patients with bacteremia in Switzerland [40] and Belgium [41], but also in samples from pig farms in Belgium [37] and in ready-to-eat food products in Poland [35]. Similarly, ST640, detected in two supermarket samples in the present study, has previously been associated with bacteremia cases in southern France [38] and Mexico [34], as well as with bovine mastitis in Korea [36] and environmental samples in Norway [42]. The ST9 lineage has mainly been associated with ocular infections [39,43]. Finally, ST59, belonging to the clinically relevant clonal complex CC2 [44,45], has been identified in companion animals and their owners [46] in addition to being detected in several human infections [34,41,44,45]. Overall, the heterogeneous population structure observed among the isolates supports the circulation of genetically diverse MRSE lineages in poultry meat rather than the dissemination of a single dominant clone. The broad distribution of these genetic lineages across food, animal, environmental, and clinical settings highlights the importance of maintaining continuous surveillance of MRSE within the food chain.

This genetic heterogeneity is in line with genomic evidence showing that *Mammaliicoccus* spp. harbor a remarkable diversity of AMR genes, including *mecA* and *blaZ*, often associated with mobile genetic elements [47]. In agreement with these findings, we detected *mecA*-positive *M. lentus* and *M. fleurettii* in poultry meat.

MDR food-borne bacteria can disseminate along processing lines, including raw products, food-contact personnel, equipment surfaces, and final products. Species-level analysis revealed a significant association between species and MDR phenotype, with *M. lentus* and *S. epidermidis* showing higher proportions of multidrug-resistant isolates, suggesting that resistance patterns are at least partially species-dependent rather than randomly distributed across the community.

Biofilm formation represents one of the most relevant virulence-associated factors in CoNS and MA, particularly in device-associated infections involving *S. epidermidis* [48]. In this study, biofilm formation was observed in 24.3% of isolates, which is a lower proportion than that reported in broiler chicken studies, where up to 80% of CoNS/MA isolates were biofilm producers [49]. Similarly, a recent study described biofilm formation in 98% of CoNS/MA isolates, although most were classified as weak producers [17].

Biofilm production in our study was also significantly associated with species, with *S. pasteuri* and *S. xylosus* exhibiting higher biofilm-forming capacity. Additional species showing biofilm production, although without statistically significant differences, included *M. lentus* (27%), *M. sciuri* (31%), *M. vitulinus* (25%), *S. chromogenes* (29%), *S. cohnii* (50%), *S. epidermidis* (22%), *S. hyicus* (20%), *S. saprophyticus* (6%), and *S. warneri* (24%). Similar findings have been reported in studies conducted in broiler chickens, where high biofilm-forming rates were observed in species such as *S. hominis* (100%), *S. xylosus* (79%), *M. sciuri* (60%), *S. simulans* (60%), and *S. saprophyticus* (47%) [36].

Additionally, the significant association between sample origin and biofilm formation, with higher biofilm production among supermarket-derived isolates, suggests that some retail environments may act as selective niches for more persistent isolates. Such conditions could facilitate bacterial survival on food-contact surfaces and contribute to contamination persistence along the food supply chain. Nevertheless, no significant correlation was detected between antibiotic resistance and biofilm formation, indicating that these traits may be independently regulated in the studied isolates.

The coexistence of antimicrobial resistance and biofilm-forming capacity in CoNS and MA represents a potential dual threat—microbial persistence and horizontal gene transfer—within food-processing environments [20]. The emergence of MDR biofilm-producing isolates poses an additional challenge with public health implications.

## 5. Conclusions

Retail chicken meat in Spain harbors diverse CoNS and *Mammaliicoccus* species that combine antimicrobial resistance and biofilm formation, constituting an under-recognized reservoir within the food chain. These results, in line with recent global genomic evidence, highlight the urgent need for integrated One Health surveillance strategies to mitigate the dissemination of AMR from food to humans.

## Figures and Tables

**Table 1:** CoNS and MA species detected in 60 poultry meat samples.

Species	Nº of Studied Isolates	Nº of Non-Repetitive Isolates	Nº of Positive Meat Samples (%)		
			Supermarkets	Local Retail Shops	Total
*S. epidermidis*	35	27 (18.2%)	6	14	20 (33.3%)
*S. simulans*	21	19 (12.8%)	7	6	13 (21.7%)
*S. saprophyticus*	36	18 (12.2%)	3	12	15 (25.0%)
*S. warneri*	19	17 (11.5%)	5	10	15 (25.0%)
*S. pasteuri*	8	8 (5.4%)	3	4	7 (11.7%)
*S. chromogenes*	7	7 (4.7%)	3	3	6 (10.0%)
*S. xylosus*	7	7 (4.7%)	2	1	3 (5.0%)
*S. hyicus*	6	5 (3.4%)	3	1	4 (6.7%)
*S. cohnii*	4	4 (2.7%)	3	1	4 (6.7%)
*S. capitis*	3	1 (0.7%)	1	0	1 (1.7%)
*M. lentus*	17	15 (10.1%)	8	7	15 (25.0%)
*M. sciuri*	14	13 (8.8%)	5	5	10 (16.7%)
*M. vitulinus*	6	4 (2.7%)	2	1	3 (5.0%)
*M. fleurettii*	3	3 (2.0%)	1	2	3 (5.0%)
Total	186	148	28	32	60

**Table 2:** Antimicrobial resistance phenotypes/genotypes and biofilm formation of the 148 non-repetitive CoNS and MA isolates.

Species (Nº of Isolates)	Resistance Phenotype ^a,b^	Resistance Genotype ^a^	Biofilm Formation ^a^
***S. epidermidis*** ** (27)**	PEN ^17^, FOX ^6^, ERY ^16^, CLI ^6^, TET ^5^, GEN ^6^, TOB ^8^, SXT ^3^, pan-susceptible ^5^	*blaZ* ^17^, *mecA* ^6^, *erm*(A) ^4^, *erm*(B) ^1^, *erm*(C) ^3^, *msr*(A) ^12^, *mph*(C) ^10^, *lnu*(A) ^1^, *vga*(A) ^2^, *tet*(K) ^4^, *tet*(L) ^4^, *tet*(M) ^2^, *aac*(6′)-Ie-*aph*(2″)-Ia ^6^, *ant*(4′)-Ia ^8^, *dfr*(A) ^3^, *dfr*(G) ^1^, *dfr*(K) ^1^	Weak ^1^, moderate ^2^, strong ^1^, very strong ^2^
***S. simulans*** ** (19)**	PEN ^6^, ERY ^1^, CLI ^3^, TET ^1^, SXT ^6^, CIP ^3^, pan-susceptible ^7^	*blaZ* ^6^, *msr*(A) ^1^, *lnu*(A) ^3^, *tet*(K) ^1^, *tet*(L) ^1^, *dfr*(A) ^5^, *dfr*(G) ^3^, *dfr*(K) ^5^	Moderate ^1^
***S. saprophyticus*** ** (18)**	TET ^6^, pan-susceptible ^12^	*tet*(K) ^6^, *tet*(L) ^5^, *tet*(M) ^2^	Moderate ^1^
***S. warneri*** ** (17)**	PEN ^9^, ERY ^7^, TET ^2^, TOB ^3^, CIP ^1^, pan-susceptible ^6^	*blaZ* ^9^, *msr*(A) ^7^, *tet*(K) ^2^, *tet*(L) ^2^, *ant*(4′)-Ia ^3^	Moderate ^3^, very strong ^1^
***S. pasteuri*** ** (8)**	PEN ^3^, ERY ^2^, CLI ^1^, TOB ^1^, pan-susceptible ^3^	*blaZ* ^4^, *msr*(A) ^2^, *lnu*(A) ^1^, *ant*(4′)-Ia ^1^	Weak ^3^, moderate ^1^, strong ^1^
***S. chromogenes*** ** (7)**	PEN ^1^, TET ^2^, CIP ^2^, pan-susceptible ^3^	*blaZ* ^2^, *mphC* ^1^, *tetK* ^2^, *tetL* ^2^	Weak ^2^
***S. xylosus*** ** (7)**	PEN ^1^, ERY ^3^, CLI ^2^, GEN ^1^, TOB ^1^, SXT ^1^, CIP ^2^, pan-susceptible ^3^	*blaZ* ^1^, *erm*(C) ^1^, *msr*(A) ^2^, *mph*(C) ^2^, *aac*(6′)-Ie-*aph*(2″)-Ia ^1^, *ant*(4′)-Ia ^1^, *dfr*(A) ^1^	Weak ^2^, moderate ^1^, very strong ^2^
***S. hyicus*** ** (5)**	FOX ^1^, CLI ^1^, CIP ^1^, pan-susceptible ^3^	*mecA* ^1^, *vga*(A) ^1^	Weak ^1^
***S. cohnii*** ** (4)**	PEN ^2^, CLI ^2^, TET ^3^, CIP ^1^, pan-susceptible ^1^	*blaZ* ^2^, *lnu*(A) ^2^, *tet*(K) ^2^, *tet*(L) ^1^, *tet*(M) ^2^	Weak ^1^, very strong ^1^
***S. capitis*** ** (1)**	Pan-susceptible	-	-
***M. lentus*** ** (15)**	PEN ^2^, FOX ^1^, ERY ^10^, CLI ^13^, TET ^8^, GEN ^3^, TOB ^6^, SXT ^8^, CIP ^5^	*blaZ* ^2^, *mecA* ^1^, *erm*(A) ^9^, *erm*(B) ^2^, *erm*(C) ^5^, *msr*(A) ^5^, *mph*(C) ^9^, *lnu*(A) ^4^, *tet*(K) ^7^, *tet*(L) ^7^, *tet*(M) ^2^, *aac*(6′)-Ie-*aph*(2″)-Ia ^3^, *ant*(4′)-Ia ^4^, *dfr*(A) ^4^, *dfr*(G) ^1^, *dfr*(K) ^7^	Weak ^2^, moderate ^2^
***M. sciuri*** ** (13)**	PEN ^2^, FOX ^1^, ERY ^6^, TET ^2^, SXT ^5^, CIP ^4^, pan-susceptible ^7^	*blaZ* ^1^, *mecA* ^1^, *erm*(A) ^2^, *erm*(B) ^1^, *erm*(C) ^1^, *msr*(A) ^6^, *mph*(C) ^3^, *tet*(K) ^2^, *tet*(L) ^1^, *dfr*(A) ^1^, *dfr*(G) ^1^, *dfr*(K) ^2^	Weak ^4^
***M. vitulinus*** ** (4)**	ERY ^1^, CLI ^1^, TET ^2^, pan-susceptible ^2^	*erm*(T) ^1^, *msr*(A) ^1^, *lnu*(A) ^1^, *tet*(K) ^2^, *tet*(L) ^2^, *tet*(M) ^1^	Moderate ^1^
***M. fleurettii*** ** (3)**	FOX ^1^, pan-susceptible ^2^	*mecA* ^1^	-

^a^ A number in superscript reflects the number of isolates that have the characteristic when not all isolates of the group have it. ^b^ PEN, penicillin; FOX, cefoxitin; ERY, erythromycin; CLI, clindamycin; TET, tetracycline; GEN, gentamicin; TOB, tobramycin; SXT, trimethoprim-sulfamethoxazole; CIP, ciprofloxacin.

## Data Availability

The original contributions presented in this study are included in the article. Further inquiries can be directed to the corresponding authors.

## Supplementary Materials

The following supporting information can be downloaded at: https://www.mdpi.com/article/10.3390/microorganisms14061195/s1, Figure S1: eBURST analysis of methicillin-resistant *S. epidermidis* isolates recovered from poultry meat samples, including allelic profiles of the detected sequence types (STs) and sample origin.

## References

[1] World Health Organization (2025). World Food Safety Day 2025 Toolkit. https://cdn.who.int/media/docs/default-source/campaigns-and-initiatives/world-food-safety-day-2025/wfsd-2025-toolkit-es.pdf.

[2] World Health Organization (2022). Food Safety. https://www.who.int/news-room/fact-sheets/detail/food-safety.

[3] Kurćubić V.S., Munjić M.D., Dmitrić M.P., Živković S., Stajić S.B., Tomasevic I. (2025). Bacterial Antimicrobial Resistance in Meat Products-Current Concepts. Foods.

[4] Haskell K.J., Schriever S.R., Fonoimoana K.D., Haws B., Hair B.B., Wienclaw T.M., Holmstead J.G., Barboza A.B., Berges E.T., Heaton M.J. (2018). Antibiotic resistance is lower in *Staphylococcus aureus* isolated from antibiotic-free raw meat as compared to conventional raw meat. PLoS ONE.

[5] Verraes C., Van Boxstael S., Van Meervenne E., Van Coillie E., Butaye P., Catry B., Herman L. (2013). Antimicrobial resistance in the food chain: A review. Int. J. Environ. Res. Public Health.

[6] Silva V., Caniça M., Ferreira E., Vieira-Pinto M., Saraiva C., Pereira J.E., Capelo J.L., Igrejas G., Poeta P. (2022). Multidrug-Resistant Methicillin-Resistant Coagulase-Negative Staphylococci in Healthy Poultry Slaughtered for Human Consumption. Antibiotics.

[7] Highmore C., Cooper K., Parker J., Robinson J., Castangia R., Webb J.S. (2025). Real-time Detection of Foodborne Pathogens and Biofilm in the Food Processing Environment with Bactiscan, A Macro-scale Fluorescence Device. J. Food Prot..

[8] Lakhundi S., Zhang K. (2018). Methicillin-Resistant *Staphylococcus aureus*: Molecular Characterization, Evolution, and Epidemiology. Clin. Microbiol. Rev..

[9] Dey S., Jana D., Manna T., Karmakar M., Paria S., Chandra Guchhait K., Hazra S., Jana P., Hossain M., Kumar Panda A. (2025). Molecular analyses of community-acquired *Staphylococcus epidermidis* biofilm development, molecular virulence, and pattern of antibiotic resistance. Mol. Biol. Rep..

[10] Fernández-Calderón M.C., Fernández-Babiano I., Navarro-Pérez M.L., Pazos-Pacheco C., Calvo-Cano A. (2025). Biofilm formation and role of other pathogenic factors in the virulence of *Staphylococcus epidermidis* clinical isolates. Front. Cell. Infect. Microbiol..

[11] Argemi X., Hansmann Y., Prola K., Prévost G. (2019). Coagulase-Negative Staphylococci Pathogenomics. Int. J. Mol. Sci..

[12] Wang Y.T., Lin Y.T., Wan T.W., Wang D.Y., Lin H.Y., Lin C.Y., Chen Y.C., Teng L.J. (2019). Distribution of antibiotic resistance genes among *Staphylococcus* species isolated from ready-to-eat foods. J. Food Drug Anal..

[13] Madhaiyan M., Wirth J.S., Saravanan V.S. (2020). Phylogenomic Analyses of the *Staphylococcaceae* Family Suggest the Reclassification of Five Species within the Genus *Staphylococcus* as Heterotypic Synonyms, the Promotion of Five Subspecies to Novel Species, the Taxonomic Reassignment of Five *Staphylococcus* Species to *Mammaliicoccus* gen. nov., and the Formal Assignment of *Nosocomiicoccus* to the Family *Staphylococcaceae*. Int. J. Syst. Evol. Microbiol..

[14] Osada M., Aung M.S., Urushibara N., Kawaguchiya M., Ohashi N., Hirose M., Kobayashi N. (2022). Prevalence and Antimicrobial Resistance of *Staphylococcus aureus* and Coagulase-Negative *Staphylococcus*/*Mammaliicoccus* from Retail Ground Meat: Identification of Broad Genetic Diversity in Fosfomycin Resistance Gene *fosB*. Pathogens.

[15] Abdullahi I.N., Latorre-Fernández J., Reuben R.C., Trabelsi I., González-Azcona C., Arfaoui A., Usman Y., Lozano C., Zarazaga M., Torres C. (2023). Beyond the Wild MRSA: Genetic Features and Phylogenomic Review of *mecC*-Mediated Methicillin Resistance in Non-*aureus* Staphylococci and Mammaliicocci. Microorganisms.

[16] Modak S., Mane P., Patil S. (2025). A Comprehensive Phenotypic Characterization of Biofilm-Producing Coagulase-Negative Staphylococci: Elucidating the Complexities of Antimicrobial Resistance and Susceptibility. Cureus.

[17] Szafraniec G.M., Chrobak-Chmiel D., Dolka I., Adamczyk K., Sułecki K., Dolka B. (2025). Virulence factors and biofilm forming ability of *Staphyloccoccus* species isolated from skeletal lesions of broiler chickens. Sci. Rep..

[18] Crippa B.L., Valente P.L.M., Barros E.L.P., Betim M.E., Almeida J.M., de Cieza M.Y.R., da Silva Pereira E., dos Santos D.L.S., Silva N.C.C. (2025). Investigation of biofilm-associated genes and biofilm formation in Non- *aureus Staphylococcus* (NAS) isolated from cow’s milk. Biofouling.

[19] Eguizábal P., Fernández-Fernández R., Campaña-Burguet A., González-Azcona C., Marañón-Clemente I., Tenorio C., Lozano C. (2024). High prevalence of avian adapted CC5 *Staphylococcus aureus* isolates in poultry meat in Spain: Food chain as vehicle of MRSA and MSSA CC398-t1451. Int. J. Food Sci. Technol..

[20] European Committee on Antimicrobial Susceptibility Testing (EUCAST) Breakpoint Tables for Interpretation of MICs and Zone Diameters. Version 12.0. http://www.eucast.org.

[21] Thomas J.C., Vargas M.R., Miragaia M., Peacock S.J., Archer G.L., Enright M.C. (2007). Improved multilocus sequence typing scheme for *Staphylococcus epidermidis*. J. Clin. Microbiol..

[22] Schwartbeck B., Birtel J., Treffon J., Langhanki L., Mellmann A., Kale D., Kahl J., Hirschhausen N., Neumann C., Lee J.C. (2016). Dynamic in vivo mutations within the *ica* operon during persistence of *Staphylococcus aureus* in the airways of cystic fibrosis patients. PLoS Pathog..

[23] Lim J.H., Park J.H., Lee G.Y., Lee J.B., Lee K.J., Yang S.J. (2025). Species Distribution, Antimicrobial Resistance, and Enterotoxin Profiles of Non-aureus Staphylococci Isolated from Poultry Slaughterhouses in Korea. Food Sci. Anim. Resour..

[24] Pyzik E., Marek A., Stȩpień-Pyśniak D., Urban-Chmiel R., Jarosz L.S., Jagiełło-Podȩbska I. (2019). Detection of antibiotic resistance and classical enterotoxin genes in coagulase -negative staphylococci isolated from poultry in Poland. J. Vet. Res..

[25] Pimenta R.L., de Melo D.A., Bronzato G.F., de Salles Souza V.R., Holmström T.C.N., de Oliveira Coelho S., de Souza M.M.S. (2021). Characterization of *Staphylococcus* spp. isolates and β-lactam resistance in broiler chicken production. Braz. J. Vet. Med..

[26] de Freitas Guimarães F., Nóbrega D.B., Richini-Pereira V.B., Marson P.M., de Figueiredo Pantoja J.C., Langoni H. (2013). Enterotoxin genes in coagulase-negative and coagulase-positive staphylococci isolated from bovine milk. J. Dairy Sci..

[27] Abdullahi I.N., Lozano C., Latorre-Fernández J., Zarazaga M., Stegger M., Torres C. (2025). Genomic analysis of multi-drug resistant coagulase-negative staphylococci from healthy humans and animals revealed unusual mechanisms of resistance and CRISPR-Cas system. Int. Microbiol..

[28] de Mesquita Souza Saraiva M., Lim K., do Monte D.F.M., Givisiez P.E.N., Alves L.B.R., de Freitas Neto O.C., Kariuki S., Júnior A.B., de Oliveira C.J.B., Gebreyes W.A. (2022). Antimicrobial resistance in the globalized food chain: A One Health perspective applied to the poultry industry. Braz. J. Microbiol..

[29] Chajęcka-Wierzchowska W., Gajewska J., Zakrzewski A.J., Caggia C., Zadernowska A. (2023). Molecular Analysis of Pathogenicity, Adhesive Matrix Molecules (MSCRAMMs) and Biofilm Genes of Coagulase-Negative Staphylococci Isolated from Ready-to-Eat Food. Int. J. Environ. Res. Public Health.

[30] Sorour H.K., Shalaby A.G., Abdelmagid M.A., Hosny R.A. (2023). Characterization and pathogenicity of multidrug-resistant coagulase-negative Staphylococci isolates in chickens. Int. Microbiol..

[31] Bhargava K., Zhang Y. (2014). Characterization of methicillin-resistant coagulase-negative staphylococci (MRCoNS) in retail meat. Food Microbiol..

[32] Singh S., Kriti M., Anamika K.S., Sharma P., Pal N., Sarma D.K., Tiwari R., Kumar M. (2025). A one health approach addressing poultry-associated antimicrobial resistance: Human, animal and environmental perspectives. Microbe.

[33] Jesumirhewe C., Odufuye T.O., Ariri J.U., Adebiyi A.A., Sanusi A.T., Stöger A., Daza-Prieto B., Allerberger F., Cabal-Rosel A., Ruppitsch W. (2024). Genetic Characterization of Antibiotic-Resistant *Staphylococcus* spp. and Mammaliicoccus sciuri from Healthy Humans and Poultry in Nigeria. Antibiotics.

[34] Martínez-Santos V.I., Torres-Añorve D.A., Echániz-Aviles G., Parra-Rojas I., Ramírez-Peralta A., Castro-Alarcón N. (2022). Characterization of *Staphylococcus epidermidis* clinical isolates from hospitalized patients with bloodstream infection obtained in two time periods. PeerJ.

[35] Podkowik M., Bystroń J., Bania J. (2012). Genotypes, antibiotic resistance, and virulence factors of staphylococci from ready-to-eat food. Foodborne Pathog. Dis..

[36] Kim S.J., Moon D.C., Park S.C., Kang H.Y., Na S.H., Lim S.K. (2019). Antimicrobial resistance and genetic characterization of coagulase-negative staphylococci from bovine mastitis milk samples in Korea. J. Dairy Sci..

[37] Argudín M.A., Vanderhaeghen W., Butaye P. (2015). Antimicrobial resistance and population structure of *Staphylococcus epidermidis* recovered from pig farms in Belgium. Vet. J..

[38] Pouget C., Chatre C., Lavigne J.P., Pantel A., Reynes J., Dunyach-Remy C. (2023). Effect of Antibiotic Exposure on Staphylococcus epidermidis Responsible for Catheter-Related Bacteremia. Int. J. Mol. Sci..

[39] Flores-Páez L.A., Zenteno J.C., Alcántar-Curiel M.D., Vargas-Mendoza C.F., Rodríguez-Martínez S., Cancino-Diaz M.E., Jan-Roblero J., Cancino-Diaz J.C. (2015). Molecular and Phenotypic Characterization of *Staphylococcus epidermidis* Isolates from Healthy Conjunctiva and a Comparative Analysis with Isolates from Ocular Infection. PLoS ONE.

[40] Dengler Haunreiter V., Boumasmoud M., Häffner N., Wipfli D., Leimer N., Rachmühl C., Kühnert D., Achermann Y., Zbinden R., Benussi S. (2019). In-host evolution of *Staphylococcus epidermidis* in a pacemaker-associated endocarditis resulting in increased antibiotic tolerance. Nat. Commun..

[41] Deplano A., Vandendriessche S., Nonhoff C., Dodémont M., Roisin S., Denis O. (2016). National surveillance of *Staphylococcus epidermidis* recovered from bloodstream infections in Belgian hospitals. J. Antimicrob. Chemother..

[42] Røken M., Iakhno S., Haaland A.H., Bjelland A.M., Wasteson Y. (2024). The Home Environment Is a Reservoir for Methicillin-Resistant Coagulase-Negative Staphylococci and Mammaliicocci. Antibiotics.

[43] Juárez-Verdayes M.A., Ramón-Peréz M.L., Flores-Páez L.A., Camarillo-Márquez O., Zenteno J.C., Jan-Roblero J., Cancino-Diaz M.E., Cancino-Diaz J.C. (2013). *Staphylococcus epidermidis* with the icaA^−^/icaD^−^/IS256^−^ genotype and protein or protein/extracellular-DNA biofilm is frequent in ocular infections. J. Med. Microbiol..

[44] Chang Y.H., Huang Y.C., Chen H.C., Ma D.H.K., Yeh L.K., Hung K.H., Hsiao C.-H. (2023). Molecular and Phenotypic Characterization of Ocular Methicillin-Resistant *Staphylococcus epidermidis* Isolates in Taiwan. Investig. Ophthalmol. Vis. Sci..

[45] Huang Y.H., Yeh Y.R., Lien R.I., Chiang M.C., Huang Y.C. (2023). Molecular characteristics and clinical features of *Staphylococcus epidermidis* healthcare-associated late-onset bacteremia among infants hospitalized in neonatal intensive care units. J. Microbiol. Immunol. Infect..

[46] Abdullahi I.N., Lozano C., González-Azcona C., Zarazaga M., Torres C. (2024). Genetic Diversification and Resistome of Coagulase-Negative Staphylococci from Nostrils of Healthy Dogs and Dog-Owners in La Rioja, Spain. Pathogens.

[47] Lienen T., Schnitt A., Hammerl J.A., Maurischat S., Tenhagen B.A. (2022). Mammaliicoccus spp. from German Dairy Farms Exhibit a Wide Range of Antimicrobial Resistance Genes and Non-Wildtype Phenotypes to Several Antibiotic Classes. Biology.

[48] Khodabux R.M.J., Mariappan S., Sekar U. (2024). Virulence, Susceptibility Profile, and Clinical Characteristics of Pathogenic Coagulase-Negative Staphylococci. Cureus.

[49] Marek A., Pyzik E., Stępień-Pyśniak D., Dec M., Jarosz Ł.S., Nowaczek A., Sulikowska M. (2021). Biofilm-Formation Ability and the Presence of Adhesion Genes in Coagulase-Negative Staphylococci Isolates from Chicken Broilers. Animals.

