# Supplementary material for: Antimicrobial Resistance and Biofilm Formation in Coagulase-Negative Staphylococcus and Mammaliicoccus spp. from Poultry Meat in Spain
Source: Microorganisms. 2026 May 26;14(6):1195. doi: 10.3390/microorganisms14061195 (PMC13303507; doi:10.3390/microorganisms14061195)
Supplement: Supplementary file 1 [file microorganisms-14-01195-s001.zip › microorganisms-4322073-supplementary.pdf]

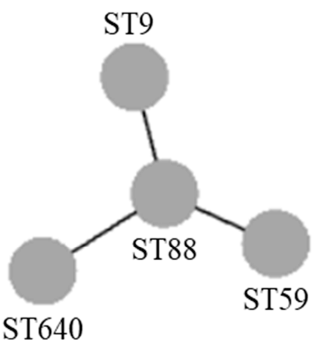

ST (nº of isolates)	Housekeeping genes							Sample Origin
	arcC	*aroE*	*gtr*	*pyrR*	*mutS*	*tpi*	*yquiL*	
**ST88 (2)**	1	1	2	1	2	1	7	Butcher retail shop
**ST640 (2)**	28	3	13	5	8	9	11	Supermarket
**ST9 (1)**	1	2	2	2	2	1	1	Butcher retail shop
**ST59 (1)**	2	1	1	1	2	1	1	Butcher retail shop

Supplementary Figure S1. eBURST analysis of methicillin-resistant *S. epidermidis* isolates recovered from poultry meat samples, including allelic profiles of the detected sequence types (STs) and sample origin.
